# Modification of Rigid Polyurethane Foams with the Addition of Nano-SiO_2_ or Lignocellulosic Biomass

**DOI:** 10.3390/polym12010107

**Published:** 2020-01-05

**Authors:** Qinqin Zhang, Xiaoqi Lin, Weisheng Chen, Heng Zhang, Dezhi Han

**Affiliations:** 1 Shandong Provincial Key Laboratory of Biochemical Engineering, College of Marine Science and Biological Engineering, Qingdao University of Science and Technology, Qingdao 266042, China; 2 State Key Laboratory Base of Eco-chemical Engineering, College of Chemical Engineering, Qingdao University of Science and Technology, Qingdao 266042, China

**Keywords:** rigid polyurethane foam, peanut shell, pine bark, nano-SiO_2_, insulation materials

## Abstract

Many achievements have been made on the research of composite polyurethane foams to improve their structure and mechanical properties, and the composite foams have been widely utilized in building insulation and furniture. In this work, rigid polyurethane foams (RPUFs) with the addition of different fillers (nano-SiO_2_, peanut shell, pine bark) were prepared through the one-step method. The effects of inorganic nano-SiO_2_ and organic biomass on foam properties were evaluated by means of physical and chemical characterization. The characterization results indicate that the compressive strength values of prepared foams could fully meet the specification requirement for the building insulation materials. The inorganic and organic fillers have no effect on the hydrogen bonding states in composite RPUFs. Furthermore, compared to the biomass fillers, the addition of nano-SiO_2_ greatly influenced the final residual content of the fabricated foam. All composite foams exhibit closed-cell structure with smaller cell size in comparison with the parent foam. The prepared composite foams have the potential for utilization in building insulation.

## 1. Introduction

In order to achieve high-quality development for current society, energy efficiency and green economy have been sustainably developing [1,2,3,4]. The construction industry is an important link to sustainable development [5]. The use of environment-friendly materials in modern buildings cannot only greatly reduce construction waste but also avoid excessive use of natural resources [6]. In view of this, the utilization of biomass, especially crop wastes, is an effective way to save energy and protect the environment in the building industry [7,8]. Furthermore, the estimated amounts of tremendous crop wastes in China covered a large scale from 620 to 940 tons every year. Therefore, the utilization of biomass resources will significantly reduce excessive CO_2_ emissions [9]. Rigid polyurethane foams (RPUFs) are the most commonly used polymer materials in the field of building for thermal insulation and sound absorption because of their preferable thermal stability; fire-, heat-, impact-, and crack-resistance; etc. [10,11,12,13,14,15]. Therefore, the research on the preparation of bio-based RPUFs or composite RPUFs with the addition of biomass fillers is necessary in future studies.

As for bio-based RPUFs, bio-based polyols, which could be produced from vegetable oils [16,17,18] and plant fibers [19,20,21] are usually served as raw materials due to the existence of abundant hydroxyl groups or double bonds in these polyols. However, chemical modification for vegetable oils or liquefaction for plant fibers is always needed to transform them into bio-based polyols, and extra energy consumption is necessary for pretreating those biomass feedstocks. Compared to bio-based RPUFs, the direct addition of biomass fillers into RPUFs has also gained much more attention. The fillers, such as nanoparticles, insulating glass microspheres, and carbon fibers, have been used to improve the functionality of polyurethane in many studies [22,23,24,25,26]. As previously reported, composite polyurethane foam with tea-leaf fibers as filler could improve sound absorption performance [27]. The electrically conductive polymer material prepared by using multiwall carbon nanotubes had a promising application in sensors and intelligent sandwich composite cores [28]. The flame-retardant property of RPUF could be enhanced by adding expanded graphite, fly ash, etc. [29,30,31,32,33]. However, many researches are still focusing on functionalization rather than sustainable development. The utilization of biomass as filler, especially crop wastes, is an effective way to fabricate RPUFs for the purpose of saving energy and protecting the environment in the construction industry to support sustainable development

The aim of this work was to synthesize the RPUFs composited with inorganic filler (nano-SiO_2_) or organic fillers (peanut shell and pine bark). Their compressive strength, hydrogen bonding states, thermal behavior, and morphology were characterized. The effects of different fillers on those physical and chemical properties of RPUFs were intensively evaluated.

## 2. Experimental

### 2.1. Materials

Polyethylene glycol 400 (PEG 400) was purchased from Tianjin Damao Chemical Co., Ltd., Tianjin, China. Polymeric methylene-4,4′-diphenyl diisocyanate (PM-200, 32.0 wt % of isocyanate group) was obtained from Wanhua Chemical Group Co., Ltd., Yantai, China. Triethylene diamine (A-33), stannous octoate (T-9), and silicone-based surfactant (L-580) were purchased from Air Products and Chemicals, Inc., Allentown, PA, USA. The hydrophilic nano-SiO_2_ with the specific surface area of 200 m^2^·g^−1^ was purchased from Aladdin biochemical technology co., Ltd., Shanghai, China. Pine bark (PB) and peanut shell (PS) were respectively obtained from Rizhao and Qingdao, China. They were dried to constant weight and then ground under the speed of 10,000 rpm. The obtained powders with the size of ≤250 μm were collected for further utilization.

### 2.2. Synthesis of Polyurethane Foam

RPUFs were synthesized via a one-step procedure. The content of all the additives was a relative weight percent to the PEG 400. Firstly, the PEG 400 (100 wt %), foaming agent (deionized water, 2.5 wt %), foam stabilizer (L-580, 2.0 wt %), catalysts (T-9 of 0.3 wt % and A-33 of 1.0 wt %), and auxiliary filler (nano-SiO_2_, PB or PS) were mixed in a 500 mL plastic beaker with stirring of 800 rpm for 5 min. Then the pre-weighted PM-200 (equivalent ratio of isocyanate group to hydroxyl group = 1/1) was quickly fed into the beaker under constant stirring. Finally, the RPUFs were demolded from beaker after 24 h curing, and then they were post-cured at room temperature for 72 h before the characterization tests. The foams composited with nano-SiO_2_, PS, and PB as filler are denoted as RPUF/SiO_2_, RPUF/PS and RPUF/PB.

### 2.3. Foams Characterization

Attenuated total reflectance Fourier transform infrared (ATR-FTIR) spectra of RPUFs were analyzed on the Bruker VERTEX 70 (Bruker Optik GmbH, Ettlingen, Germany) with 32 scans under the frequency range of 4000–400 cm^−1^ and a resolution of 2 cm^−1^. The thermogravimetry (TG) scans were collected on a Netzsch Simultaneous Thermal Analyzer STA 449F5 Jupiter (Netzsch, Selb, Germany) from 30 to 800 °C at the heating rate of 10 °C·min^−1^ under the nitrogen atmosphere. The apparent densities of RPUFs were measured according to GB/T 6343-2009. The compressive strength was tested according to GB/T 8813-2008 on an electronic universal testing machine of H10KS (Hounsfield Test Equipment Ltd., Redhill, Surrey City, UK) at a loading speed of 5 mm·min^−1^. The foam cell structure was observed through a cold-field emission scanning electron microscope of Hitachi S-4800 (Hitachi High-Technologies Corp., Tokyo, Japan).

## 3. Result and Discussion

### 3.1. Compressive Strength

**Figure 1:**
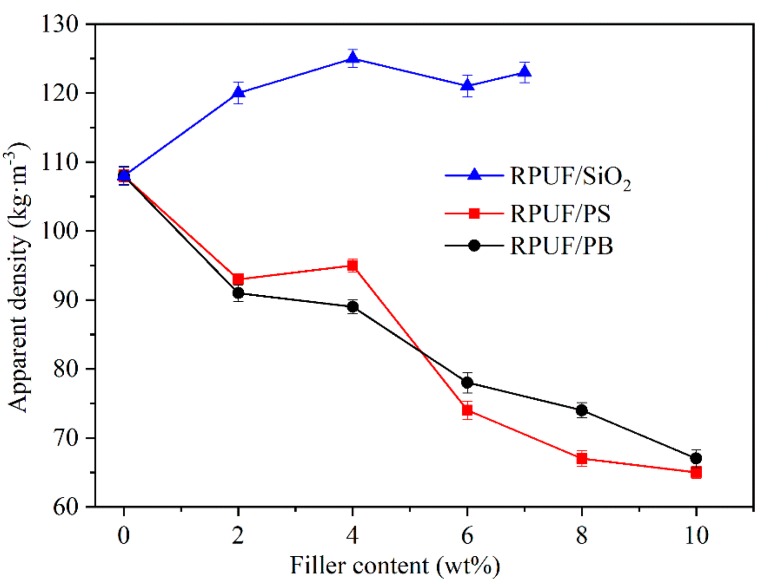
The apparent density of prepared composite RPUFs.

Compressive strength, which depends greatly on the foam density, is the crucial parameter to evaluate the compression properties of RPUFs. The specific compressive strength (the ratio of compressive strength to density) was used in this study for characterizing the compression properties of the prepared composite forms [34]. It can be seen from Figure 1 that the apparent density increases with the increase in nano-SiO_2_ content (up to 4 wt %), then almost reaches a plateau. However, when the addition amount of nano-SiO_2_ was >7 wt %, the viscosity of blends experiences a sharp increase, resulting in the shutdown of the foaming reaction [35]. In the case of the addition of lignocellulosic biomass into the RPUF foaming process, the density of obtained foams decreases with the elevated content of PS or PB. The lignocellulosic biomass filler could act as the nucleating agent for promoting foam growing process to rapidly form a low-density foam.

**Figure 2:**
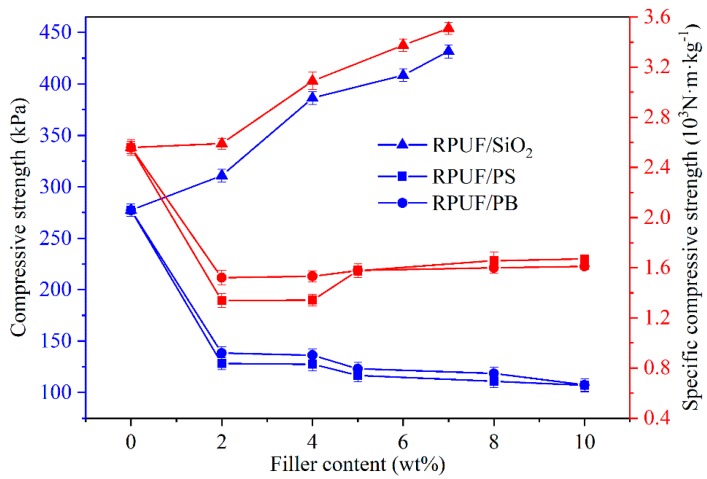
The compressive strength and specific compressive strength curves of prepared composite RPUFs under the deformation degree of 10%.

As illustrated in Figure 2, the compressive strength has the same change tendency as the specific compressive strength of prepared composite RPUFs. Their values increase with the increase of nano-SiO_2_ content in the RPUF/SiO_2_ samples. The presence of nano-SiO_2_ would lead to the formation of thick and dense pore walls, thus could improve the compressive strength of RPUF/SiO_2_ samples. However, the compressive strength and specific compressive strength of RPUF/PS or RPUF/PB samples represent a rapid decrease with the addition of PS or PB (≤2%), then varies slightly with further increase in PS or PB content. This could be attributed to the loose pores arrangement and relatively thin pore walls in those foams, which would be also discussed in the following discussion of the morphology investigation. These results indicated that the type and the addition content of fillers could significantly tune the physical properties of the fabricated RPUFs as compared to the previous reports [36,37]. According to GB/T 21558-2008, the compressive strength value of RPUFs used as the building insulation materials should be higher than 80 kPa. Therefore, the compressive strength of the above composite RPUFs meets the requirement for external wall insulation in building fields.

### 3.2. Hydrogen Bonding States from ATR-FTIR

**Figure 3:**
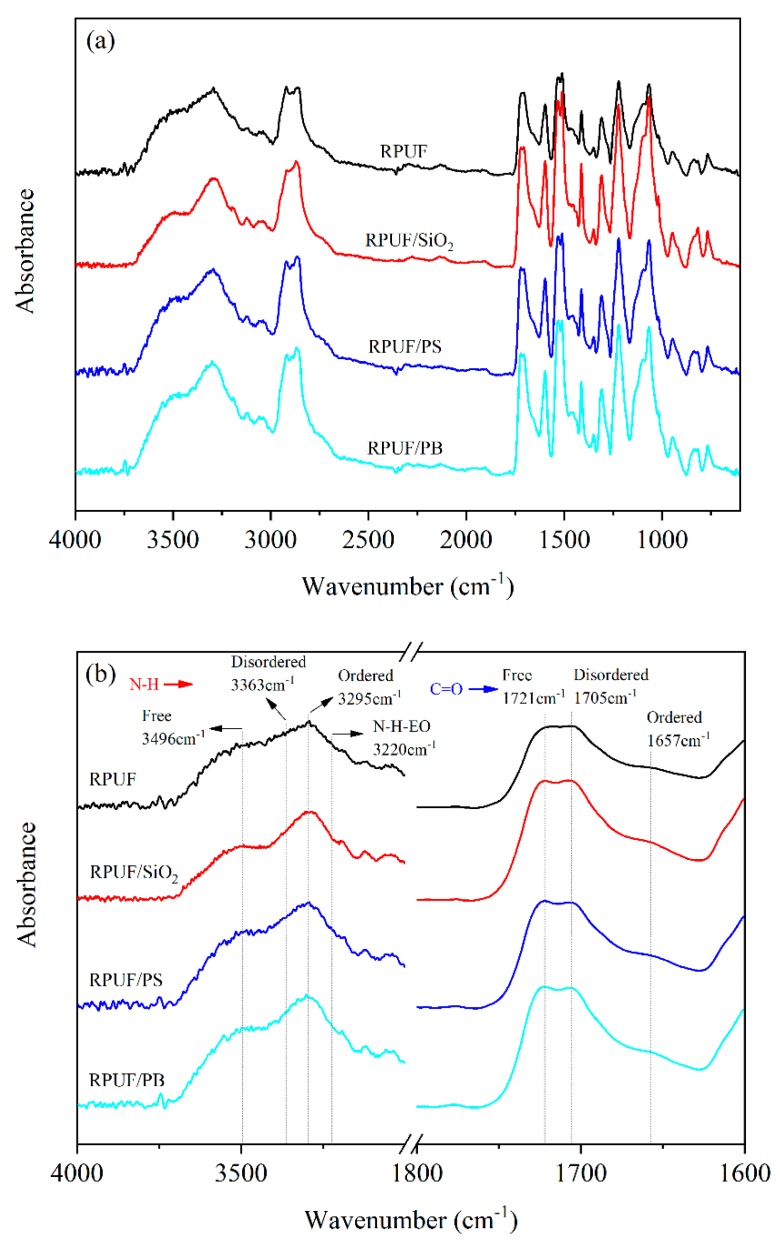
The ATR-FTIR spectra of RPUFs with and without fillers: integrated curves (**a**) and partially enlarged curves (**b**).

The ATR-FTIR is generally used for characterizing the hydrogen bonding (H-bonding) states in polyurethanes. Figure 3 shows the integrated (a) and partial (b) ATR-FTIR spectra of RPUFs with and without the addition of fillers. It can be seen from Figure 3a that all the prepared RPUFs exhibit the similar spectra, and there is no stretching vibration peak at around 2275 cm^−1^ for isocyanate (N=C=O) group, demonstrating that the isocyanate groups have reacted completely with polyols and water during polymerization [38]. Furthermore, two principal vibration regions for N–H stretching and C=O stretching band, with free (non-H-bonding), ordered (strongly H-bonding) and disordered (amorphous) states, can be observed in Figure 3b. Apparently, the ordered and disordered N–H stretching vibrations appear at around 3363 cm^−1^ and 3295 cm^−1^, respectively. The free N–H stretching vibration is present at 3496 cm^−1^ as a shoulder peak on the side of those bands [39]. The hydrogen bonding of N–H with ether oxygen (N−H−EO) is responsible for the absorbance peak at 3220 cm^−1^. Moreover, the characteristic absorbance peaks of H-bonding C=O groups generally appear at lower wavenumbers [40]. The stretching vibration peaks located at 1721 cm^−1^, 1705 cm^−1^, and 1657 cm^−1^ could be ascribed to the free, disordered and ordered hydrogen bonding state in the C=O groups, respectively [41,42]. These results imply that the organic or inorganic fillers have no effect on the hydrogen bonding states in composite RPUFs in comparison with the parent RPUF. The fillers did not react with the ingredients in the foaming process, but participated as inert materials to only change the physical properties of the prepared composite RPUFs.

### 3.3. Thermal Behavior

**Figure 4:**
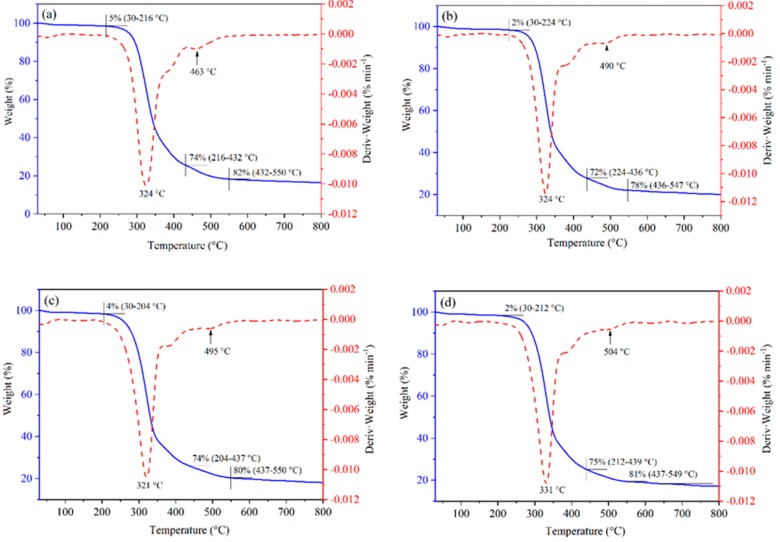
The TG and DTG curves of (**a**) RPUF, (**b**) RPUF/SiO_2_ (7 wt %), (**c**) RPUF/PS (10 wt %), and (**d**) RPUF/PB (10 wt %).

The thermal stability of polyurethanes is related to the intrinsic nature as well as the equivalent ratio of functional groups of the hard and soft segment. Moreover, the degree of phase separation between the hard and soft segments also plays a considerable role in determining the thermal stability of polyurethanes [43]. The thermal behavior of the prepared RPUFs was characterized by thermogravimetry (TG) and the corresponding differential thermogravimetry (DTG), as depicted in Figure 4. Compared to parent RPUF, there are no obvious changes in TG curve shape after the addition of nano-SiO_2_, PS or PB. The substantial degradation stage above 200 °C is attributed to the urethane bond decomposition through the breakdown of isocyanate and polyols [44], forming the primary amines and terminal olefinic group on the polyester chain [45], and the maximum weight loss rate can be observed at around 320–330 °C from DTG curves. Moreover, a weak weight loss peak can be found at the higher temperature (430–550 °C), probably resulting from the C–C bond cleavage [46], and the final residual content for RPUF, RPUF/SiO_2_, RPUF/PS and RPUF/PB is 16.4%, 20.0%, 18.0%, and 17.1%, respectively. The thermal stability of RPUFs is improved by the addition of the fillers, especially the inorganic one. The organic fillers could be decomposed when heated, while the inorganic nano-SiO_2_ filler remained stable, thus leading to the high residual content of the RPUF/SiO_2_.

### 3.4. Morphology Investigation

**Figure 5:**
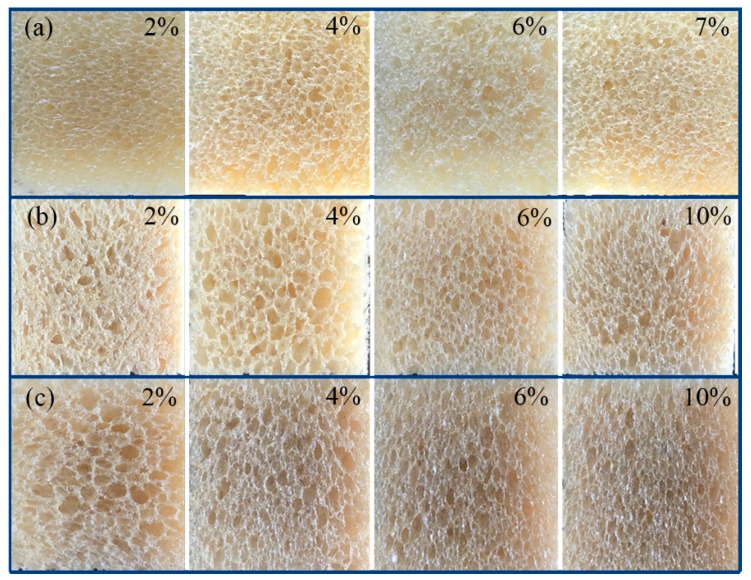
Camera pictures of (**a**) RPUF/SiO_2_ (7 wt %), (**b**) RPUF/PS (10 wt %), and (**c**) RPUF/PB (10 wt %).

**Figure 6:**
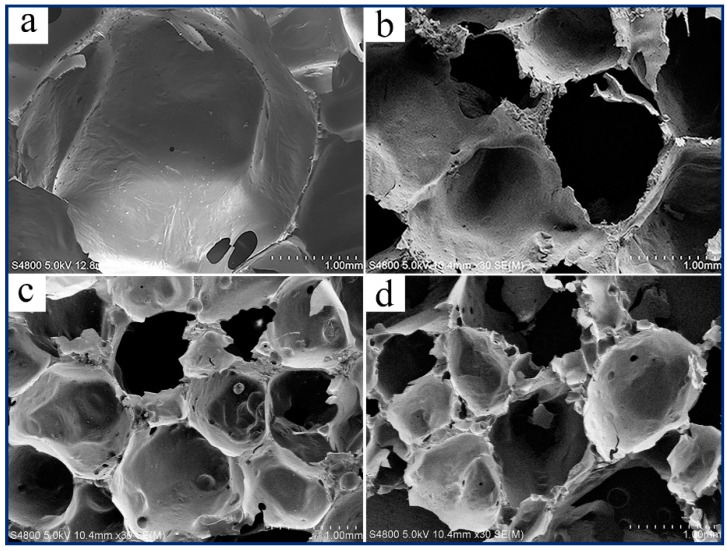
The SEM images of (**a**) RPUF, (**b**) RPUF/SiO_2_ (7 wt %), (**c**) RPUF/PS (10 wt %), and (**d**) RPUF/PB (10 wt %).

The camera pictures and SEM images of prepared RPUFs are shown in Figure 5 and Figure 6, respectively, to investigate the foam structure such as pore size, cell walls, and wall joints. Morphology changes of RPUFs mainly depend on the preparation condition of samples. It can be seen from Figure 5 that the cell size of RPUF/SiO_2_ is smaller than that of the RPUF/PS and RPUF/PB, which is consistent with the results of the apparent density analysis (Figure 1). RPUFs samples for SEM observation were cut from the perpendicular orientation to the foam growth direction. It can be observed from Figure 6 that all prepared RPUFs possess the closed-cell structure with many windows, and the cell size of composite RPUFs with the addition of fillers is much smaller than that of the parent foam. The closed-cell structure of the prepared composite RPUFs could be beneficial for the application of acoustical and thermal insulation.

## 4. Conclusions

In this work, the effects of inorganic and organic fillers on compressive strength, density, thermal behavior, and morphology of rigid polyurethane foams were investigated. The results indicated the density of RPUF/SiO_2_ increased with the increase in the filler content while the density of RPUF/PS and RPUF/PB experienced the opposite trend. The compressive strength values of prepared RPUFs could fully meet the specification requirement for the building insulation materials. Moreover, the organic or inorganic fillers have no effect on the hydrogen bonding states in composite RPUFs in comparison with the parent RPUF. The addition of inorganic nano-SiO_2_ filler could bring relatively higher residual content in the corresponding RPUF/SiO_2_, while the organic fillers could be easily decomposed when heated, leading to the lower residual content of the RPUF/PS and RPUF/PB. The close-cell structure and appropriate compressive strength make the composite RPUFs suitable for serving as building insulation materials.

## References

[1] The G20 Seoul Summit Leaders’ Declaration. http://www.g20.utoronto.ca/.

[2] Hwang B.-G., Zhao X., See Y.L., Zhong Y. (2015). Addressing Risks in Green Retrofit Projects: The Case of Singapore. Proj. Manag. J..

[3] Ravindu S., Rameezdeen R., Zuo J., Zhou Z., Chandratilake R. (2015). Indoor environment quality of green buildings: Case study of an LEED platinum certified factory in a warm humid tropical climate. Build. Environ..

[4] Roetzel A., Tsangrassoulis A., Dietrich U. (2014). Impact of building design and occupancy on office comfort and energy performance in different climates. Build. Environ..

[5] Wolfson A., Litvak G., Dlugy C., Shotland Y., Tavor D. (2009). Employing crude glycerol from biodiesel production as an alternative green reaction medium. Ind. Crops Prod..

[6] Sricharoenchaikul V., Atong D. (2012). Fuel Gas Generation from Thermochemical Conversion of Crude Glycerol Mixed with Biomass Wastes. Energy Procedia.

[7] Briga-Sá A., Nascimento D., Teixeira N., Pinto J., Caldeira F., Varum H., Paiva A. (2013). Textile waste as an alternative thermal insulation building material solution. Constr. Build. Mater..

[8] Rajput D., Bhagade S.S., Raut S.P., Ralegaonkar R.V., Mandavgane S.A. (2012). Reuse of cotton and recycle paper mill waste as building material. Constr. Build. Mater..

[9] Liu H., Jiang G., Zhuang H., Wang K. (2008). Distribution, utilization structure and potential of biomass resources in rural China: With special references of crop residues. Renew. Sustain. Energy Rev..

[10] Sung G., Kim J.W., Kim J.H. (2016). Fabrication of polyurethane composite foams with magnesium hydroxide filler for improved sound absorption. J. Ind. Eng. Chem..

[11] Wang Y., Zhang C., Ren L., Ichchou M., Galland M.A., Bareille O. (2013). Influences of rice hull in polyurethane foam on its sound absorption characteristics. Polym. Compos..

[12] Tiuc A.E., Vermeşan H., Gabor T., Vasile O. (2016). Improved Sound Absorption Properties of Polyurethane Foam Mixed with Textile Waste. Energy Procedia.

[13] Luo F., Wu K., Lu M. (2016). Enhanced thermal stability and flame retardancy of polyurethane foam composites with polybenzoxazine modified ammonium polyphosphates. RSC Adv..

[14] Shimizu T., Koshiro S., Yamada Y., Tada K. (1997). Effect of cell structure on oil absorption of highly oil absorptive polyurethane foam for on-site use. J. Appl. Polym. Sci..

[15] Zhou X., Zhang Z., Xu X., Men X., Zhu X. (2013). Facile Fabrication of Superhydrophobic Sponge with Selective Absorption and Collection of Oil from Water. Ind. Eng. Chem. Res..

[16] Kurańska M., Prociak A. (2016). The influence of rapeseed oil-based polyols on the foaming process of rigid polyurethane foams. Ind. Crops Prod..

[17] Kairytė A., Vėjelis S. (2015). Evaluation of forming mixture composition impact on properties of water blown rigid polyurethane (PUR) foam from rapeseed oil polyol. Ind. Crops Prod..

[18] Bähr M., Mülhaupt R. (2012). Linseed and soybean oil-based polyurethanes prepared via the non-isocyanate route and catalytic carbon dioxide conversion. Green Chem..

[19] Zhang G., Wu Y., Chen W., Han D., Lin X., Xu G., Zhang Q. (2019). Open-Cell Rigid Polyurethane Foams from Peanut Shell-Derived Polyols Prepared under Different Post-Processing Conditions. Polymers.

[20] Zhang G., Zhang Q., Wu Y., Zhang H., Cao J., Han D. (2017). Effect of auxiliary blowing agents on properties of rigid polyurethane foams based on liquefied products from peanut shell. J. Appl. Polym. Sci..

[21] Xie J., Zhai X., Hse C.Y., Shupe T.F., Pan H. (2015). Polyols from Microwave Liquefied Bagasse and Its Application to Rigid Polyurethane Foam. Materials.

[22] Şerban D.A., Weissenborn O., Geller S., Marşavina L., Gude M. (2016). Evaluation of the mechanical and morphological properties of long fibre reinforced polyurethane rigid foams. Polym. Test..

[23] Akkoyun M., Suvaci E. (2016). Effects of TiO_2_, ZnO, and Fe_3_O_4_ nanofillers on rheological behavior, microstructure, and reaction kinetics of rigid polyurethane foams. J. Appl. Polym. Sci..

[24] Yakushin V., Bel’kova L., Sevastyanova I. (2012). Properties of rigid polyurethane foams filled with glass microspheres. Mech. Compos. Mater..

[25] Yakushin V., Stirna U., Bel’kova L., Deme L., Sevastyanova I. (2011). Properties of rigid polyurethane foams filled with milled carbon fibers. Mech. Compos. Mater..

[26] Mukherji A., Mishra S. (2007). Effect of Sizes of Nano Ca_3_(PO_4_)_2_ on Mechanical and Thermal Properties of Polyurethane Foam Composites. Polym. Plast. Technol. Eng..

[27] Ekici B., Kentli A., Küçük H. (2012). Improving Sound Absorption Property of Polyurethane Foams by Adding Tea-Leaf Fibers. Arch. Acoust..

[28] Athanasopoulos N., Baltopoulos A., Matzakou M., Vavouliotis A., Kostopoulos V. (2012). Electrical conductivity of polyurethane/MWCNT nanocomposite foams. Polym. Compos..

[29] Gama N.V., Silva R., Mohseni F., Davarpanah A., Amaral V.S., Ferreira A., Barros-Timmons A. (2018). Enhancement of physical and reaction to fire properties of crude glycerol polyurethane foams filled with expanded graphite. Polym. Test..

[30] Tarakcılar A.R. (2011). The effects of intumescent flame retardant including ammonium polyphosphate/pentaerythritol and fly ash fillers on the physicomechanical properties of rigid polyurethane foams. J. Appl. Polym. Sci..

[31] Wolska A., Goździkiewicz M., Ryszkowska J. (2012). Thermal and mechanical behaviour of flexible polyurethane foams modified with graphite and phosphorous fillers. J. Mater. Sci..

[32] Wang X., Shi Y., Liu Y., Wang Q. (2019). Recycling of waste melamine formaldehyde foam as flame-retardant filler for polyurethane foam. J. Polym. Res..

[33] Yao Y.Y., Tian H.F., Yuan L., Wu Q.X., Xiang A.M. (2018). Improved mechanical, thermal, and flame-resistant properties of polyurethane-imide foams via expandable graphite modification. J. Appl. Polym. Sci..

[34] Zhou W., Bo C., Jia P., Zhou Y., Zhang M. (2018). Effects of Tung Oil-Based Polyols on the Thermal Stability, Flame Retardancy, and Mechanical Properties of Rigid Polyurethane Foam. Polymers.

[35] Javni I., Zhang W., Karajkov V., Petrovic Z.S., Divjakovic V. (2016). Effect of Nano-and Micro-Silica Fillers on Polyurethane Foam Properties. J. Cell. Plast..

[36] Członka S., Strąkowska A., Strzelec K., Kairytė A., Vaitkus S. (2019). Composites of rigid polyurethane foams and silica powder filler enhanced with ionic liquid. Polym. Test..

[37] Kairytė A., Kizinievič O., Kizinievič V., Kremensas A. (2019). Synthesis of biomass-derived bottom waste ash based rigid biopolyurethane composite foams: Rheological behaviour, structure and performance characteristics. Compos. Part A: Appl. Sci. Manuf..

[38] Bolcu C., Vlase G., Vlase T., Albu P., Doca N., Şisu E. (2013). Thermal behavior of some polyurethanes reticulated by aminated maltose. J. Therm. Anal. Calorim..

[39] Vander Schuur M., Noordover B., Gaymans R.J. (2006). Polyurethane elastomers with amide chain extenders of uniform length. Polymer.

[40] Yu T.L., Lin T.L., Tsai Y.M., Liu W.J. (1999). Morphology of polyurethanes with triol monomer crosslinked on hard segments. J. Polym. Sci. Part B Polym. Phys..

[41] Sormana J.L., Meredith J.C. (2004). High-Throughput Discovery of Structure—Mechanical Property Relationships for Segmented Poly(urethane—urea)s. Macromolecules.

[42] Hernandez R., Weksler J., Padsalgikar A., Runt J. (2007). Microstructural Organization of Three-Phase Polydimethylsiloxane-Based Segmented Polyurethanes. Macromolecules.

[43] Chattopadhyay D.K., Webster D.C. (2009). Thermal stability and flame retardancy of polyurethanes. Prog. Polym. Sci..

[44] Petrović Z.S., Zavargo Z., Flyn J.H., Macknight W.J. (1994). Thermal Degradation of Segmented Polyurethanes. J. Appl. Polym. Sci..

[45] Levchik S.V., Weil E.D. (2004). Thermal decomposition, combustion and flame-retardancy of epoxy resins? A review of the recent literature. Polym. Int..

[46] Kong X., Narine S.S. (2007). Physical properties of polyurethane plastic sheets produced from polyols from canola oil. Biomacromolecules.

